# Prenatal diagnosis of BACs‐on‐Beads assay in 1520 cases from Fujian Province, China

**DOI:** 10.1002/mgg3.1446

**Published:** 2020-08-07

**Authors:** Yan Wang, Min Zhang, Lingji Chen, Hailong Huang, Liangpu Xu

**Affiliations:** ^1^ Fujian Key Laboratory for Prenatal Diagnosis and Birth Defect, Fujian Maternity and Child Health Hospital, Affiliated Hospital of Fujian Medical University Fuzhou China

**Keywords:** BoBs™, CMA, FISH, karyotyping, microdeletions, microduplications, PND

## Abstract

**Background:**

The aim of this study was to evaluate the application of BACs‐on‐Beads (BoBs™) assay for rapid detection of chromosomal abnormalities for prenatal diagnosis (PND).

**Methods:**

A total of 1520 samples, including seven chorionic villi biopsy samples, 1328 amniotic fluid samples, and 185 umbilical cord samples from pregnant women were collected to detect the chromosomal abnormalities using BoBs™ assay and karyotyping. Furthermore, abnormal specimens were verified by chromosome microarray analysis (CMA) and fluorescence in situ hybridization (FISH).

**Results:**

The results demonstrated that the success rate of karyotyping and BoBs™ assay in PND was 98.09% and 100%, respectively. BoBs™ assay was concordant with karyotyping for Trisomy 21, Trisomy 18, and Trisomy 13, sex chromosomal aneuploidy, Wolf–Hirschhorn syndrome, and mosaicism. BoBs™ assay also detected Smith–Magenis syndrome, Williams–Beuren syndrome, DiGeorge syndrome, Miller–Dieker syndrome, Prader–Willi syndrome, Xp22.31 microdeletions, 22q11.2, and 17p11.2 microduplications. However, karyotyping failed to show these chromosomal abnormalities. A case of 8q21.2q23.3 duplication which was found by karyotyping was not detected by BoBs™ assay. Furthermore, all these chromosomal abnormalities were consistent with CMA and FISH verifications. According to the reports, we estimated that the detection rates of karyotyping, BoBs™, and CMA in the present study were 4.28%, 4.93%, and 5%, respectively, which is consistent with the results of a previous study. The respective costs for the three methods were about $135–145, $270–290, and $540–580.

**Conclusion:**

BoBs™ assay is considered a reliable, rapid test for use in PND. A variety of comprehensive technological applications can complement each other in PND, in order to maximize the diagnosis rate and reduce the occurrence of birth defects.

## Graphical abstract

BoBs™ assay has its unique advantages. Detection rates of Karyotype, BoBs™, and CMA were 4.41%, 4.93%, and 5% in our study, 2%–4%, 3%–5%, and 4%–7% in previous study. Costs of the three methods were about 13–5145$, 270–290$, and 540–580$.

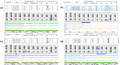

## 1 INTRODUCTION

Prenatal diagnosis (PND) is a technique employed to detect the chromosomal abnormalities in fetuses before birth, which is an important means of reducing birth defects (Alesi, Bertoli, Sinibaldi, & Novelli, 2013). Karyotyping is the gold standard for PND and can detect aneuploidies and large structural chromosome rearrangements (>5 Mb) (Yi et al., 2019). With the advent of molecular cytogenetic technologies such as fluorescence in situ hybridization (FISH) and chromosome microarray analysis (CMA), it is now understood that chromosomal microdeletions and microduplications below the karyotyping resolution contribute significantly to diseases (Cui et al., 2019; Quintela et al., 2015; Yi et al., 2019). The application of various molecular diagnostic techniques to detect microdeletions and microduplications plays a critical role in PND (Chen et al., 2014; Karcaaltincaba et al., 2010; Klugman et al., 2014). BACs‐on‐Beads (BoBs™) assay was modified from comparative genomic hybridization and developed to detect the DNA copy number gains and losses. BoBs™ assay is a bead‐based multiple assay using beads impregnated with two different fluorochromes of unlike concentrations to create an array of up to 100 different unique probes, each probe is derived from a BAC DNA that can allow the diagnosis of common abnormalities and nine microdeletions within 30 h (García‐Herrero et al., 2014). Here, we report that BoBs™ assay has unique application advantages in PND.

## 2 MATERIALS AND METHODS

### 2.1 Ethical compliance

The study design and protocol were reviewed and approved by the ethics committee of Fujian Maternity and Child Health Hospital (No. 2016021). All procedures performed in these studies involving human participants were conducted in accordance with the ethical standards of the institutional and/or national research committee and with the 1964 Helsinki declaration and its later amendments or comparable ethical standards. Informed consent was obtained from all individual participants included in the study.

### 2.2 Study design

1520 samples were collected between July 2017 and June 2019 in Fujian Maternity and Child Health Hospital (Fujian, China) and included seven chorionic villi biopsy samples (7/1520,0.46%), 1328 amniotic fluid samples (1328/1520,87.37%), and 185 umbilical cord centesis samples (185/1520, 12.17%). Pregnant women from 18 to 43 years of age with an average age of 24.63 ± 2.47 years were considered. According to the clinical indications for invasive PND, detailed characteristic of these gravid women are displayed in Table 1, including simple advanced age (*n* = 506), high risk of NIPT (*n* = 31), high‐risk pregnancy of serological screening in early and middle stages (*n* = 315), abnormal ultrasound (*n* = 398), adverse pregnancy history (*n* = 35), two kinds of abnormal indications (*n* = 198), three kinds of abnormal indications (*n* = 19), and others categories (*n* = 18) assessed in this study.

**Table 1:** Reasons for referral and anomaly rate of specimens

Clinical indications	Total	Normal	Abnormal	Anomaly rate (%)
Simple advanced age	506	485	21	4.15
High risk of NIPT	31	29	2	6.45
High‐risk pregnancy of serological screening	315	300	15	4.76
Abnormal ultrasound	398	375	23	5.78
Adverse pregnancy history	35	33	2	5.71
Two kinds of abnormal indications	198	187	11	5.56
Three or more kinds of abnormal indications	19	17	2	10.53
Others	18	17	1	5.56

### 2.3 DNA isolation

Genomic DNA was extracted using a commercially available DNA extraction kit (Qiagen, Hilden, Germany) according to the manufacturer's protocols. The extracted DNA was quantified by a Nanodrop 2000 Spectrophotometer (Thermo Fisher Scientific, MA, USA). Finally, the genomic DNA was stored at −80°C until further use.

### 2.4 Karyotyping

G‐banded karyotyping was performed according to the standard protocols in our laboratory at the 320–500 bands level. Karyotyping was determined according to the International System for Human Cytogenetic Nomenclature 2016 (ISCN 2016).

### 2.5 BoBs^™^ assay

BoBs™ assay can detect the copy number changes of chromosomes 13, 18, 21, X, and Y as well as nine microdeletion syndromes. The nine microdeletion syndromes analyzed were Wolf–Hirschhorn syndrome (WHS), Cri du Chat syndrome, Williams–Beuren syndrome (WBS), Langer–Giedion syndrome, Prader–Willi/Angelman syndrome (PWS/AGS), Miller–Dieker syndrome (MDS), Smith–Magenis syndrome (SMS), and DiGeorge syndrome (DGS). Critical regions of the nine microdeletion syndromes are shown in Table 2. This assay was obtained from BoBs™ assay manufacturer (PerkinElmer, Wallac Oy, Finland), and the fluorescence data were analyzed with BoBsoft software (PerkinElmer, Wallac Oy, Finland). The samples were defined as deleted/duplicated at a specific chromosomal locus when the ratios of the fluorescence intensities fell outside the threshold of the mean ± 2 SDs, typically ranging between 0.6 and 0.8 (deleted) and between 1.3 and 1.4 (duplicated), respectively (Vialard et al., 2012).

**Table 2:** Critical regions of the nine microdeletion syndromes detected by BoBs™

Syndrome	Target region
Wolf–Hirschhorn	4p16.3
Cri du Chat	5p15.2–5p15.3
Williams–Beuren	7q11.2
Langer–Giedion	8q23–8q24
Prader–Willi/Angelman	15q11–15q12
Miller–Dieker	17p13.3
Smith–Magenis	17p11.2
DiGeorgeⅠ	22q11.2
DiGeorgeⅡ	10p14

### 2.6 Chromosome microarray analysis

The microdeletions or microduplications detected by BoBs™ assay were then validated with CMA using the Thermo Fisher CytoScan 750 K arrays (Thermo Fisher Scientific, MA, USA) according to the manufacturer's protocols. Data were analyzed using Chromosome Analysis Suite software 3.1.

### 2.7 Fluorescence in situ hybridization

FISH assay was performed on one case using an AneuVysion Assay Kit (Abbott Park, Illinois, USA). The D18Z1, DXZ1, and DYZ3 probes were selected based on the gain region detected by CMA. Metaphase FISH analysis was performed on the amniocytes according to the manufacturer's protocol.

### 2.8 Statistical analysis

Statistical analyses were performed using SPSS 18.0 (IBM, USA). Data were presented as the mean ± SD (standard deviation) between three independent experiments with each being measured in triplicate. The differences among the groups were analyzed using One‐way ANOVA. A value of *p* < 0.05 was considered being a statistically significant difference.

## 3 RESULTS

### 3.1 Abnormal rates of different clinical indications

According to the primary clinical diagnosis, the 1520 samples were categorized into eight groups (Table 1). Anomaly rate for each clinical indication was as follows: simple advanced age (21/506,4.15%), high risk of NIPT (2/31,6.45%), high‐risk pregnancy serological screening (15/315,4.76%), abnormal ultrasound (23/398,5.78%), adverse pregnancy history (2/35,5.71%), two kinds of abnormal indications (11/198, 5.56%), three or more kinds of abnormal indications (2/19,10.53%), and others (1/18, 5.56%). Except for three or more kinds of abnormal indications had higher rates, there was no significant difference in abnormal detection rates among the other groups (*p* > 0.05).

### 3.2 Common aneuploidies involving Chromosomes 13, 18, and 21, and sex chromosomes

**Figure 1:**
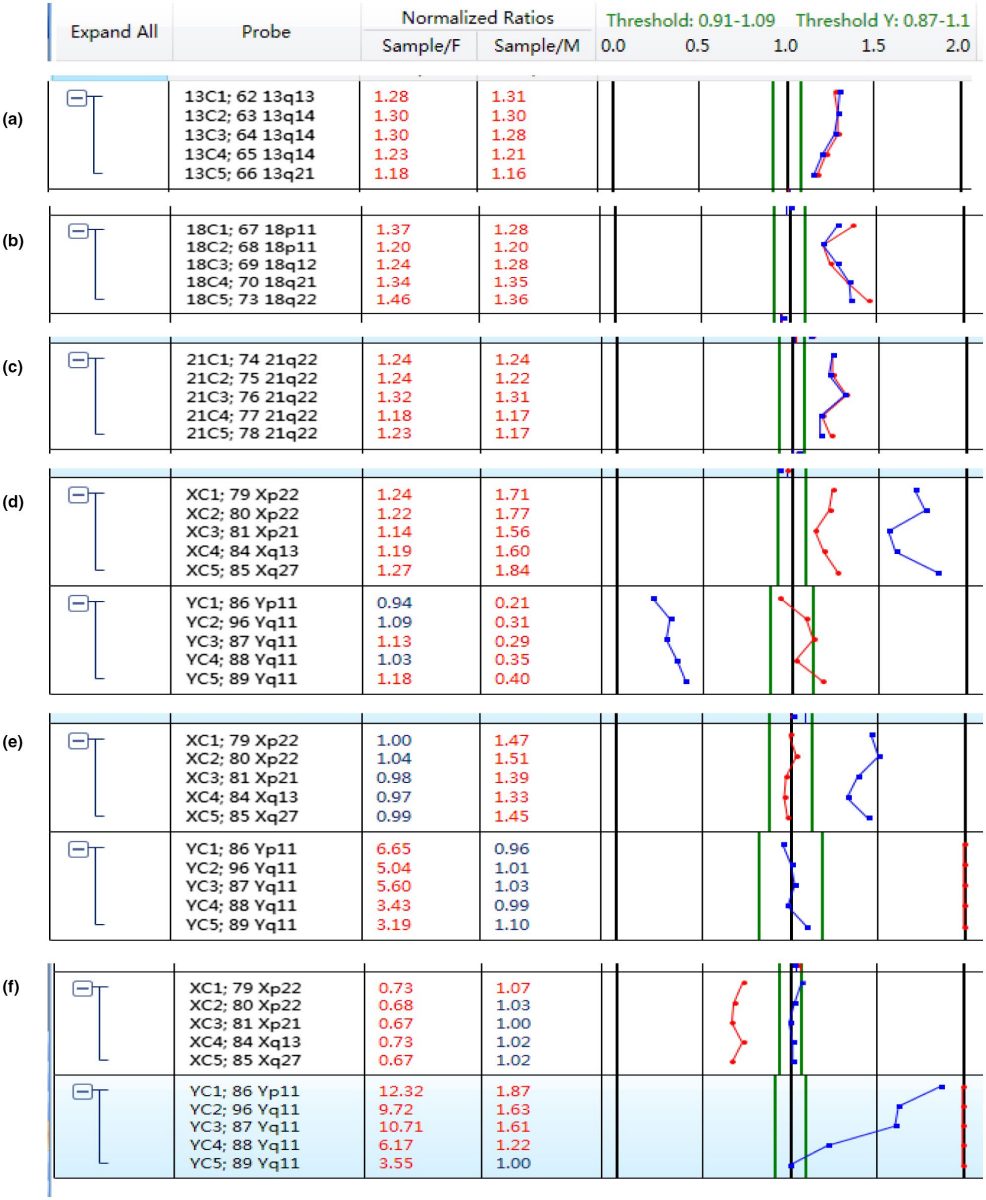
The maps of aneuploidies involving Chromosomes 13, 18, and 21 and the sex chromosomes to which BoBs™ assay was applied. (a) Trisomy 13. (b) Trisomy 18. (c) Trisomy 21. (d) XXX. (e) XXY. (f) XYY. The blue dots represent the proportion of tested DNA compared with the male reference DNA. The red dots represent the proportion of tested DNA compared with female reference DNA. The green lines are at the normal signal range. 93 × 46 mm (300 × 300 DPI)

Karyotyping and BoBs™ assay were applied to detect chromosomal abnormalities from a total number of 1520 samples. The success rate of karyotyping for PND was 98.09% (1491/1520), while the success rate of BoBs™ assay was 100% (1520/1520) (Table 3). BoBs™ assay was concordant with karyotyping for Trisomy 21(1.78%, 27/1520), Trisomy 18 (0.72%, 11/1520), Trisomy13 (0.26%, 4/1520), sex chromosomal aneuploidy (1.12%, 17/1520), including seven cases of 47,XYY, six cases of 47,XXX, and four incidents of 47,XXY (Table 4). BoBs™ assay maps of aneuploidies involving Chromosomes 13, 18, and 21, and the sex chromosomes are found in Figure 1.

**Table 3:** Comparison of karyotyping and BoBs™

Methods	Success	Failure	Normal	Abnormal	Success rate
Karyotyping	1491	29	1426	65	98.01% (1491/1520)
BoBs™	1520	0	1445	75	100% (1520/1520)

**Table 4:** Detailed chromosomal abnormalities and pregnancy outcome detected by karyotyping, BoBs™, CMA, and FISH.

Chromosome abnormality	Cases	Could be detected by:				Fetal outcome
		Karyotyping	BoBs™	CMA	FISH	
Trisomy 21	27	27	27	–	**–**	TOP
Trisomy 18	11	11	11	**–**	**–**	TOP
Trisomy 13	4	4	4	**–**	**–**	TOP
47, XYY	7	7	7	**–**	**–**	TOP
47, XXX	6	6	6	**–**	**–**	TOP
47, XXY	4	4	4	**–**	**–**	TOP
SMS	1	0	1	1	**–**	TOP
WHS	1	1	1	1	**–**	TOP
WBS	1	0	1	1	**–**	TOP
DGS	3	0	3	3	**–**	TOP
MDS	2	0	2	2	**–**	TOP
PWS	1	0	1	1	**–**	TOP
Xp22.31 microdeletions	1	0	1	1	**–**	Normal
22q11.2 microduplications	1	0	1	1	**–**	Normal
17p11.2 microduplications	1	0	1	1	**–**	TOP
8q21.2q23.3 duplication	1	1	0	1	**–**	TOP
mosaicism	4	4	4	3	1	TOP
Total	76	65	75	16	1	**–**

Abbreviations: DGS, DiGeorge syndrome; MDS, Miller–Dieker syndrome; PWS, Prader–Willi/Angelman syndrome; SMS, Smith–Magenis syndrome; TOP, terminate of pregnancy; WBS, Williams–Beuren syndrome; WHS, Wolf–Hirschhorn syndrome.

### 3.3 Chromosomal deletions and duplications

**Figure 2:**
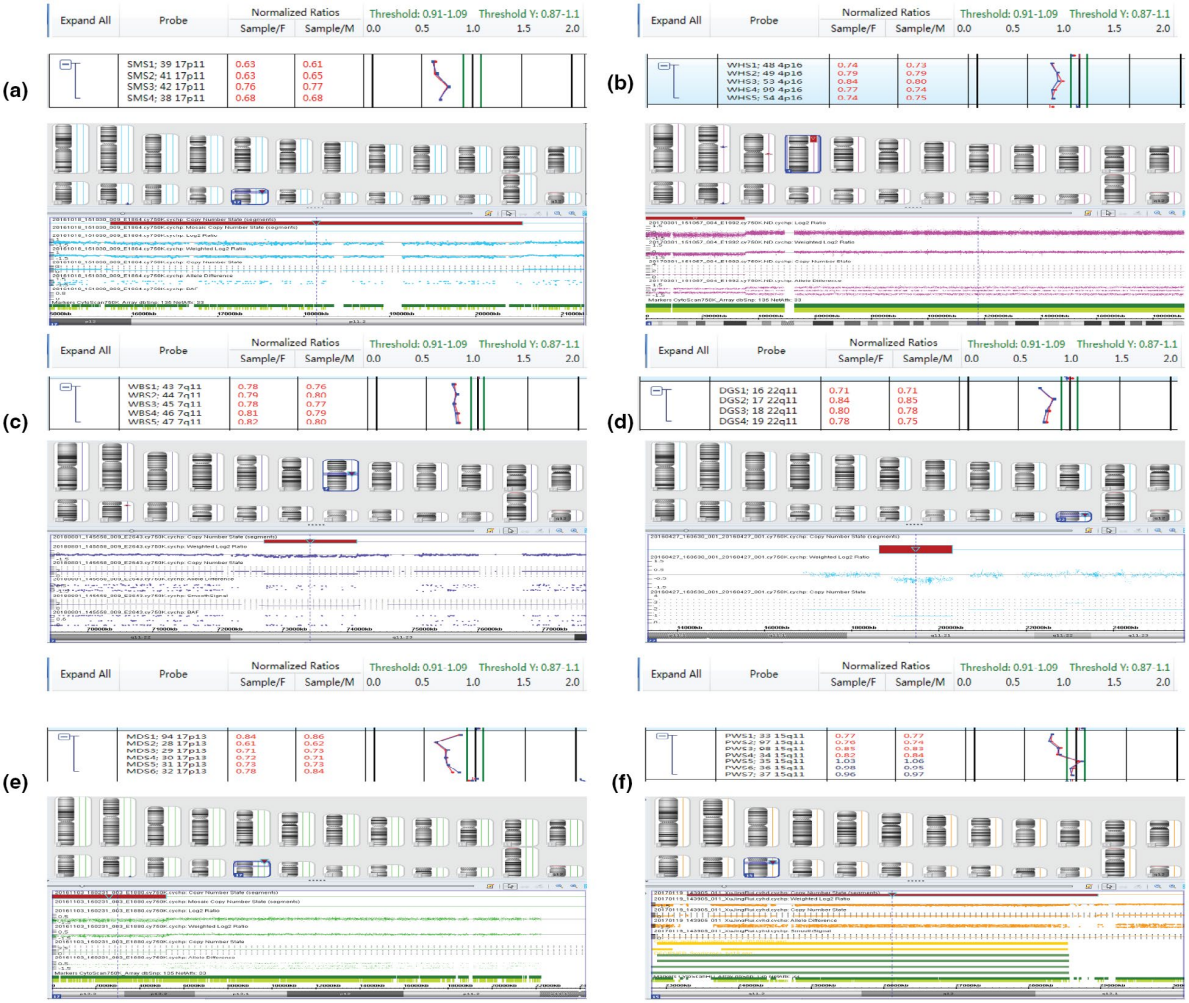
The maps within the detection range of BoBs™ assay. (a) SMS deletion syndrome (4.78 Mb lost in 17p11.2). (b) WHS deletion syndrome (35 Mb lost in 4p16.3p15.1). (c) WBS deletion syndrome (1.4 Mb lost in 7q11.23). (d) DGS deletion syndrome (1.8 Mb lost in 21q11.2). (e) MDS deletion syndrome (5.2 Mb lost in 17p11.3). (f) PWS deletion syndrome (5.7 Mb lost in 15q11.2q13.1). CMA: The red lines represent lost; the blue lines represent gain

**Figure 3:**
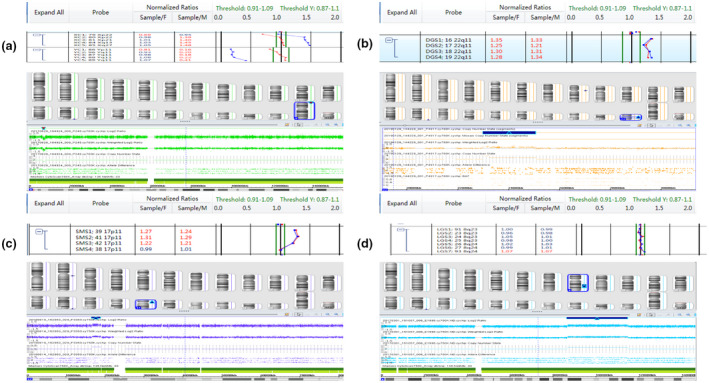
The maps outside of the detection range of BoBs™ assay. (a) Xp22.31 microdeletions (1.2 Mb). (b) 22q11.2 microduplications (2.0 Mb) (c) 17p11.2 microduplications (4.8 Mb). (d) 8q21.2q23.2 duplication (28 Mb)

BoBs™ assay detected one case of WHS deletion syndrome (35 Mb lost in 4p16.3p15.1), consisting with karyotyping results. BoBs™ assay also detected one case of SMS microdeletions, one case of WBS microdeletions, three cases of DGS microdeletions, two cases of MDS microdeletions, one case of PWS microdeletions, one case of Xp22.31 microdeletions, one case of 22q11.2 microduplications, and one case of 17p11.2 microduplications, whereas karyotyping failed to detect these chromosomal abnormalities. A case of 8q21.2q23.3 duplication which was found by karyotyping was not detected by BoBs™ assay. Furthermore, all these chromosomal abnormalities are consistent with CMA verification. BoBs™ and CMA maps of deletions and duplications are found in Figures 2 and 3.

### 3.4 Mosaicism

**Figure 4:**
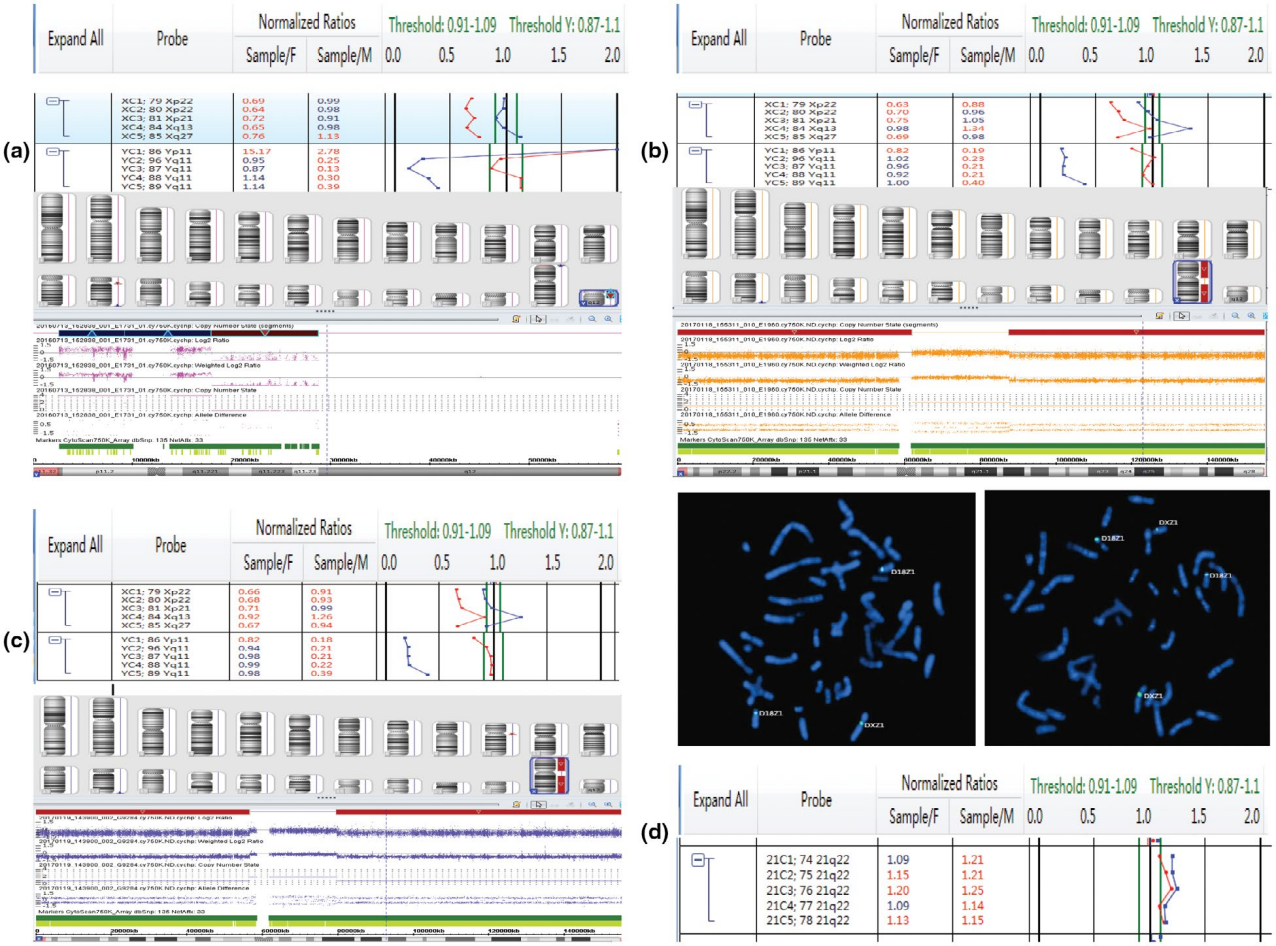
Mosaicism maps employing BoBs™, CMA, and FISH assays. (a) Yp11.31q11.21 duplication (15.3 Mb) and Yq11.221q11.23 deletion (10.7 Mb). (b) Xp22.33q11.21 deletion (62 Mb) and Xq21.31q28 deletion (68 Mb). FISH using DXZ1 and D18Z1 probes for the X and 18 chromosomes centromeres (green and turquoise signals, respectively). (c) Xp22.33q11.1 deletion (56 Mb) and Xq21.1q28 deletion (75 Mb). (d) Trisomy21 (mosaicism)

Four cases of mosaicism were identified by both prenatal BoBs™ and karyotyping. One case with small supernumerary marker chromosomes was verified by CMA and FISH (Table 5). Two cases with small supernumerary marker chromosomes were verified by CMA only. BoBs™, CMA, and FISH maps of mosaicism are shown in Figure 4.

**Table 5:** Mosaicism detected by karyotyping, BoBs™, CMA, and FISH.

Type	Karyotyping	BoBs™	CMA	FISH
CB	46, X, +mar [86]/45, X [14] (86%/14%)	Detected	arr[hg19] Yp11.31q11.221(2,650,424–18,016,216) ×4	–
			Yq11.221q11.23(18,047,379–28,799,654) ×0	
CB	46, X, +mar [21]/45, X [12] (63.64%/36.36%)	Detected	arr[hg19] Xp22.33p11.21(168,551–56,661,860) ×1	46, X, mar.ish r(X)(DXZ1+) [8]/45, X [6]
			Xq21.1q28(79,764,187–155,233,098) ×1	
AF	46, X, +mar [22]/45, X [20] (52.38%/47.62%)	Detected	arr[hg19] Xp22.33q11.1(168,551–62,006,469) ×1	–
			Xq21.31q28(87,685,781–155,233,098) ×1	
AF	47, XY, +21[37]/46, XY [62] (37.37%/62.63%)	Detected	–	–

Abbreviations: AF, Amniotic fluid; CB, Cord blood.

### 3.5 Pregnancy outcomes

Of the 1520 prenatal cases collected, 1428 cases were successfully followed up, while 92 cases were lost. The follow‐up rate was 93.95% (1428/1520). Of the 76 abnormal cases detected, 74 cases were terminated and two had normal deliveries. Among 1352 cases with normal chromosomes, 79 cases were terminated due to obvious abnormalities in ultrasound, and 1273 cases had normal deliveries.

### 3.6 Comparison

According to the reports, CMA has been 100% accurate in identifying common aneuploidies as compared to karyotyping. It also can detect mosaicisms of over 30% abnormal cells (Breman et al., 2012; Callaway, Shaffer, Chitty, Rosenfeld, & Crolla, 2013). We estimated the detection rates of karyotyping, BoBs™, and CMA in the present study to be 4.28% (65/1520), 4.93% (75/1520), and 5% (76/1520) (Table 6); 2%–4% (Chen et al., 2017; Li, Chen, et al., 2019); 3%–5% (Chen et al., 2017; Fang et al., 2018; Li, Chen, et al., 2019) and 4%–7% (Srebniak et al., 2012, 2016) in a previous study. The respective costs of the three methods were approximately $135–145, $270–290, and $540–580.

**Table 6:** Detection rates and costs of each method: karyotyping, BoBs™, and CMA

Methods	Cost	Detection rates	
		Present study	Previous study
Karyotyping	135–145$	4.28% (65/1520)	2%–4%^10,11^
BoBs™	270–290$	4.93% (75/1520)	3%–5%^10−12^
CMA	540–580$	5% (76/1520)	4%–7%^13,14^

## 4 DISCUSSION

Chromosomal abnormality is the main reason for congenital anomalies. It is estimated that six percent of congenital defects are due to aneuploidies and nearly one in 200 newborns is affected. Karyotyping is routinely used for PND of chromosomal abnormalities such as aneuploidy and balanced translocations. However, it cannot detect deletions and duplications of small fragments of less than 5 Mb (Yi et al., 2019). In addition, it has many disadvantages, including the need for cell culture, greater manual operation time, long reporting cycles (the average reporting time is 14 days), and the possibility of cultivating failure. Prenatal BoBs™ technology can detect most common aneuploidies involving chromosomes 13, 18, 21, X, and Y as well as nine microdeletion syndromes found at high incidence in human population (the accumulative incidence can reach 1/1700) (Shaffer & Van den Veyver, 2012) without cell culture. Furthermore, BoBs™ assay has the advantage of short detection period (it can be completed within 30 h), simple operation procedure, high screening rate (92 samples can be detected at one time), intuitive detection results, and easy interpretation (Vialard et al., 2011). Therefore, we should balance the benefits and drawbacks of conventional karyotyping against BoBs™ assay.

The results of our retrospective study on 1520 samples from Fujian province in China have provided further assurance on the increased diagnostic yield of BoBs™ assay compared with karyotyping. The chorionic villi biopsy, amniotic fluid, and umbilical cord centesis samples were cultured, and conventional karyotyping was performed in parallel. The success rate of BoBs™ assay was higher than that of karyotyping (100% vs. 98.09%). Around 27 cases of Trisomy 21, 11 cases of Trisomy 18, 4 cases of Trisomy 13, and 17 cases of sex chromosomal aneuploidy were detected by both karyotyping and BoBs™ assay. BoBs™ assay detected one case of WHS syndrome that was verified by CMA and was consistent with karyotyping results. The missing fragments above were 35 MB, covering all areas of the syndrome. Based on our data, the application of BoBs™ assay in PND is very trustworthy and advocating involved multicenter research (Vialard et al., 2011, 2012).

BoBs™ assay also detected three cases of DGS microdeletions, two cases of MDS microdeletions, one case of SMS microdeletions, one case of WBS microdeletions, one case of PWS microdeletions, one case of Xp22.31 Microdeletions, one case of 22q11.2 microduplications, and one case of 17p11.2 microduplications, whereas karyotyping failed to reveal these chromosomal abnormalities. Furthermore, all these chromosomal abnormalities were verified by CMA. Microduplications were not the scope of the BoBs™ report, but the results can also have a certain role in suggesting the involvement of a microduplication syndrome, while additional prompts can improve detection rates. Submicroscopic deletions at Xp22.31 lead to the loss of *ST*S (MIM: 300747) and *ANOS1* (MIM: 300836) genes, which can cause X‐linked ichthyosis and Kallmann syndrome, respectively (Nagai et al., 2017; Quintela et al., 2015). Male patients with X‐linked ichthyosis also exhibits severe hypogenitalism and hypogonadism (Nagai et al., 2017). The case with Xp22.31 microdeletions detected in the study was a female fetus carrier of ichthyosis without skin abnormalities followed up at 6 months post‐partum. The chromosome 22q11.2 deletion has been implicated in genomic diseases for long (Stachon et al., 2007). Chromosome duplications of the region in patients have been reported, establishing a new genomic duplication syndrome (Hoeffding et al., 2017; Li, Yi, et al., 2019; Portnoi, 2009; Vaisvilas et al., 2018) complementary to the 22q11.2 deletion syndrome. In the study, one fetus with 22q11.2 microduplications was identified who showed ventricular defect. The pregnancy was terminated. Potocki–Lupski syndrome (PTLS) occurs in approximately 1 in 25,000 births and is associated with congenital anomalies and intellectual disability (Popowski et al., 2012). PTLS is caused by genetic duplication within 17p11.2 region (Potocki et al., 2000, 2007). The fetus with 17p11.2 microduplications detected in the study had cardiovascular abnormalities and similarly the pregnancy was terminated. As long as the area covered by BoBs™ probe can be detected, BoBs™ assay can offer additional diagnostic benefit compared with karyotyping and provide greater sensitivity for detecting microdeletions and microduplications (García‐Herrero et al., 2014; Gross et al., 2011).

One case involving 8q21.2q23.3 duplication was found by karyotyping but was not detected by BoBs™ assay. CMA showed an increased copy number of the pathogenic genome in chromosome 8q21.2q23.3 with copy number three and a fragment size of about 28 Mb. This increased region contains multiple pathogenic OMIM genes that can cause growth retardation, mental retardation, craniofacial deformities, multiple malformations, and congenital anomalies such as heart and kidney malformations (Piotrowski et al., 2014; Tsang, Yang, & Fong, 2014). The fetus with 8q21.2q23.3 duplication had cardiovascular abnormalities and the pregnancy was terminated. Since the added area was not within the BoBs™ probe coverage, the result of BoBs™ assay was normal. The limited detection range of BoBs™ assay reduces the detection of abnormal rate.

Mosaicism was observed in 0.8%–1.5% of prenatal samples (Hoang et al., 2011). At present, the most commonly used method to assess mosaicism is karyotyping. Its accuracy is depended not only on the proportion of abnormal cells present but also on the number of cells being analyzed (Chen et al., 2016). The ability of prenatal BoBs™ assay to detect chromosome mosaicism has been explored with a 20% to 30% ratio of mosaicism (Cheng et al., 2013). In our study, four cases of mosaicism at different ratios were detected, the lowest of which was 14%. This showed that the ability of BoBs™ assay to detect chromosome mosaicism was beyond our previous expectations. For those three cases of sex chromosome mosaicism, BoBs™ assay was only able to detect sex chromosome abnormality. Karyotype analysis can only determine the ratio of mosaicism, while CMA and FISH can clarify the sources of small supernumerary marker chromosomes. Of the three small marker chromosomes, two were parts of X chromosome and one was part of Y. The combined analysis of multiple detection methods can better explain the results. BoBs™ assay and CMA were based on uncultured cells, which can reflect the fetal situation more realistically. PND of 45,X/46,XY mosaicism occurs approximately in 1.7 per 10,000 prenatal samples with phenotype ranging from 90% of normal male fetuses to postnatal features that include a wide spectrum of phenotypes such as hypospadias (Barone et al., 2011). PND plays an important role in preventing the transmission of genetic abnormalities. All the cases of mosaicism in our study informed choices to terminate pregnancies.

Based on our data, the anomaly rate of simple advanced age, high risk of NIPT, high‐risk pregnancy of serological screening, abnormal ultrasound, adverse pregnancy history, and other factors was not significantly different (*p* > 0.05). PND of all high‐risk pregnant women is very necessary.

The respective detection rates of karyotyping, BoBs™ and CMA were 4.28%, 4.93%, and 5% in the present study; 2%–4% (Chen et al., 2017; Li, Yi, et al., 2019), 3%–5% (Chen et al., 2017; Fang et al., 2018; Li, Chen, et al., 2019), and 4%–7% (Srebniak et al., 2012, 2016) in a previous study. The respective costs of the three methods were about $135–145, $270–290, and $540–580. Although molecular diagnostics technologies are constantly emerging and changing, BoBs™ assay has unique advantages in terms of its detection rate and cost‐effectiveness.

Our study exhibited some limitations. Samples with common aneuploidies were not detected by CMA as improving test results. The study lacked multicenter collaboration and large amount of data. Thus, further studies with multicenter surveys and larger sample sizes are required to confirm our findings.

In conclusion, BoBs™ assay is considered a reliable, rapid test for PND. A variety of comprehensive technological applications can complement one another in PND in order to maximize the diagnosis rate and reduce the occurrence of birth defects.

## CONFLICT OF INTEREST

The authors declare that no conflict of interest was present that can be perceived as having prejudiced the impartiality of the research reported.

## AUTHOR'S CONTRIBUTION

Study concepts: Yan Wang, Data collection and analysis: Min Zhang, Lingji Chen, Yan Wang, Manuscript editing: Yan Wang, Manuscript revision/review: Yan Wang, Hailong Huang, Manuscript final version approval: Liangpu Xu.
